# Oblique Lumbar Interbody Fusion Using a Stand-Alone Construct for the Treatment of Adjacent-Segment Lumbar Degenerative Disease

**DOI:** 10.3389/fsurg.2022.850099

**Published:** 2022-04-01

**Authors:** Wang Kai, Cheng Cheng, Qingyu Yao, Can Zhang, Fengzeng Jian, Hao Wu

**Affiliations:** Department of Neurosurgery, Xuanwu Hospital of Capital Medical University, Beijing, China

**Keywords:** oblique lumbar interbody fusion, stand-alone, adjacent-segment disease, visual analog scale, Oswestry Disability Index

## Abstract

**Objective:**

Adjacent-segment disease (ASD) is common in patients undergone previous lumbar fusion. A typical revision treatment from posterior approach requires management of postoperative scar tissue and previously implanted instrumentation. An oblique lumbar interbody fusion (OLIF) approach allows surgeon to reduce the potential risk of posterior approach. This study aimed to analyze the clinical and radiographic efficacy of stand-alone OLIF for the treatment of lumbar adjacent-segment disease.

**Methods:**

A total of 13 consecutive patients who underwent stand-alone OLIF for the treatment of adjacent-segment disease from December 2016 to January 2019 were reviewed. Visual analog scale (VAS) of back pain and leg pain and the Oswestry Disability Index (ODI) before surgery and at last postoperative clinic visits were obtained. Radiography, CT and MRI before and at last follow-up after surgery was evaluated in all patients.

**Results:**

During the study period, 13 cases were successfully treated with stand-alone OLIF. The mean follow-up was 17.7 ± 8.3 months. The back pain VAS improved from 6.2 ± 1.0 to 2.0 ± 1.1 (*P* < 0.01), and the leg pain VAS improved from 7.0 ± 1.9 to 1.0 ± 0.9 (*P* < 0.01). ODI improved from 28.0 ± 7.5 to 10.8 ± 4.0 (*P* < 0.01). The disc height (DH) increased from 9 ± 2 to 12 ± 2 mm (*P* < 0.01), the cross-sectional area (CSA) of spinal canal increased from 85 ± 26 to 132 ± 24 mm^2^ (*P* < 0.01), the foraminal height increased from 17 ± 2 to 21 ± 3 mm (*P* < 0.01) and the CSA of foramen increased from 95 ± 25 to 155 ± 36 mm^2^ (*P* < 0.01). Cage subsidence was observed in 2 cases.

**Conclusions:**

Stand-alone OLIF provides a safe and effective alternative way to treat ASD.

## Introduction

Adjacent-segment disease (ASD) is a common phenomenon following lumbar spinal fusion (1). The development of adjacent segment disease is undoubtedly multifactorial. While some studies attribute this to increased motion and biomechanical forces on the unfused segments, it is also clear that patient characteristics, including age, sex and previously lumbar degeneration could predispose the patient to further degeneration (2–5). Many studies have shown that the rate of ASD is ~3% per year (6). The patients developing ASD experience axial pain, radiculopathy or neurogenic claudication (3, 7).

Operative management to achieve decompression and stabilization for symptomatic ASD should be considered after failure of non-operative management (6). Traditionally, this is performed posteriorly with laminectomy, extension of the instrumentation and fusion level. However, this method requires extensive soft tissue dissection to expose the previously implanted hardware, adding to prolonged operation time, blood loss, postoperative pain and prolonging recovery with high associated health care costs (3, 8, 9). In addition, exposing the previous laminectomy site poses a higher risk of dural violations and Cerebrospinal Fluid (CSF) leakage due to postoperative scar tissue (3, 10). As minimally invasive lateral lumbar interbody fusion has become increasingly popular over the past decade, it offers the surgeon an alternative strategy for revision surgery to avoid these risks (3). It allows the surgeon to achieve indirect decompression and interbody fusion. The effectiveness of indirect decompression relies on distraction across the intervertebral space to stretch the spinal ligaments and enlarge the central canal as well as increase the foraminal space for the exiting nerve root (11–13). However, the transpsoas approach is associated with direct muscle injury and a risk of injury to the lumbar plexus as it courses through the psoas (14–16). The oblique lumbar interbody fusion (OLIF) was introduced as an alternative procedure to the transpsoas approach, allowing for psoas preservation and avoids the lumbar plexus (17, 18). Several studies have reported promising results of OLIF for primary surgery of lumbar degenerative disease (12, 13, 18–20). However, there are few reports of stand-alone OLIF for treatment of ASD (21). The radiographic indirect decompression effect of spinal canal and foramen, namely the CSA of spinal canal and foramen, has not been evaluated yet.

Here, we report a consecutive series of patients who had undergone stand-alone OLIF without additional instrumentation to achieve indirect decompression and stabilization for ASD and evaluate the clinical and radiographic efficacy of this approach.

## Patient and Methods

### Study Population

This is a retrospective study. A total of 13 consecutive patients who had undergone stand-alone OLIF for ASD after lumbar fusion in Xuanwu Hospital between December 2016 and January 2019 were included in this study based on the following inclusion criteria: (I) clinical and radiographic findings as reported by Cheh et al. were consistent with progressive degeneration at the adjacent spinal level with associated new back and/or leg symptoms (2); (II) refractory to conservative measures including NSAIDs and epidural injection; (II) single-level stand-alone OLIF for the treatment of ASD to a lumbar fusion construct. Patients were excluded from the study if they had undergone surgery for a non-degenerative etiology such as infection or trauma. Medical records, operative reports and radiographic imaging studies were retrospectively reviewed.

### Surgical Technique

A stand-alone procedure was defined by the absence of instrumentation at the OLIF level. The OLIF procedures were performed using the OLIF (DePuy Synthes, Raynham, MA, USA), as similarly described in previous reports (18). The patient was put in the right lateral decubitus position with spine flex to increase the distance between the iliac crest and the rib cage. A 4-cm skin incision was made about 5 cm anterior to the mid portion of an intervertebral disc of interest, parallel to the fibers of the external oblique. The retroperitoneal space is accessed by blunt dissection. The peritoneal content was mobilized anteriorly and the psoas muscle was retracted posteriorly, revealing the intervertebral disc. After confirming the segment of intervertebral disc with fluoroscopy, we incised the annulus, remove the disc material with curettes and rongeurs and prepare the endplates. An appropriately sized polyetheretherketone (PEEK) cage (DePuy Synthes, Raynham, MA, USA) is then filled with allogeneic bone graft and hydroxyapatite containing bone morphogenetic protein (BMP) mixed with bone marrow which is aspirated from iliac crest. Neither posterior fixation nor lateral fixation was applied. All patients were allowed to ambulate by Boston brace on the second postoperative day. The Boston brace was recommended for removal after 12 weeks.

### Outcome Measures

**Figure 1:**
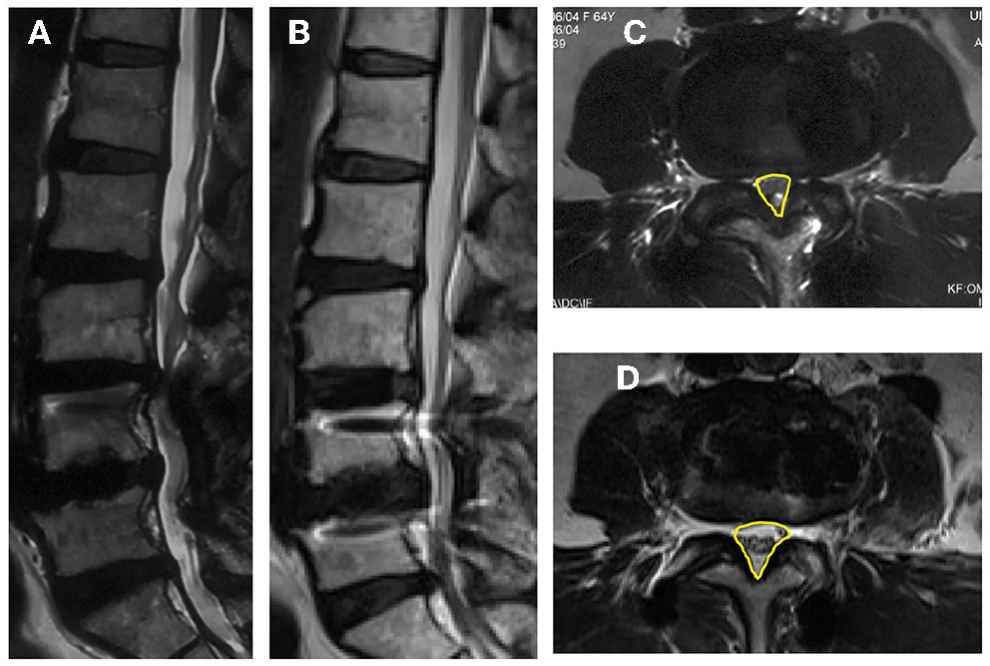
Images obtained in a 62-year-old woman who had an L4–L5 posterior instrumented fusion 6 years earlier. She experienced new back and leg pain and intermittent claudication due to adjacent-segment degeneration and stenosis for 3 months. **(A)** Pre-operative sagittal MRI; **(B)** Sagittal MRI at last follow-up post-operative; **(C)** Pre-operative axial MRI through the L3–4 and CSA (the yellow contour line illustrates); **(D)** Post-operative axial MRI through the L3–4 and CSA (the yellow contour line illustrates).

**Figure 2:**
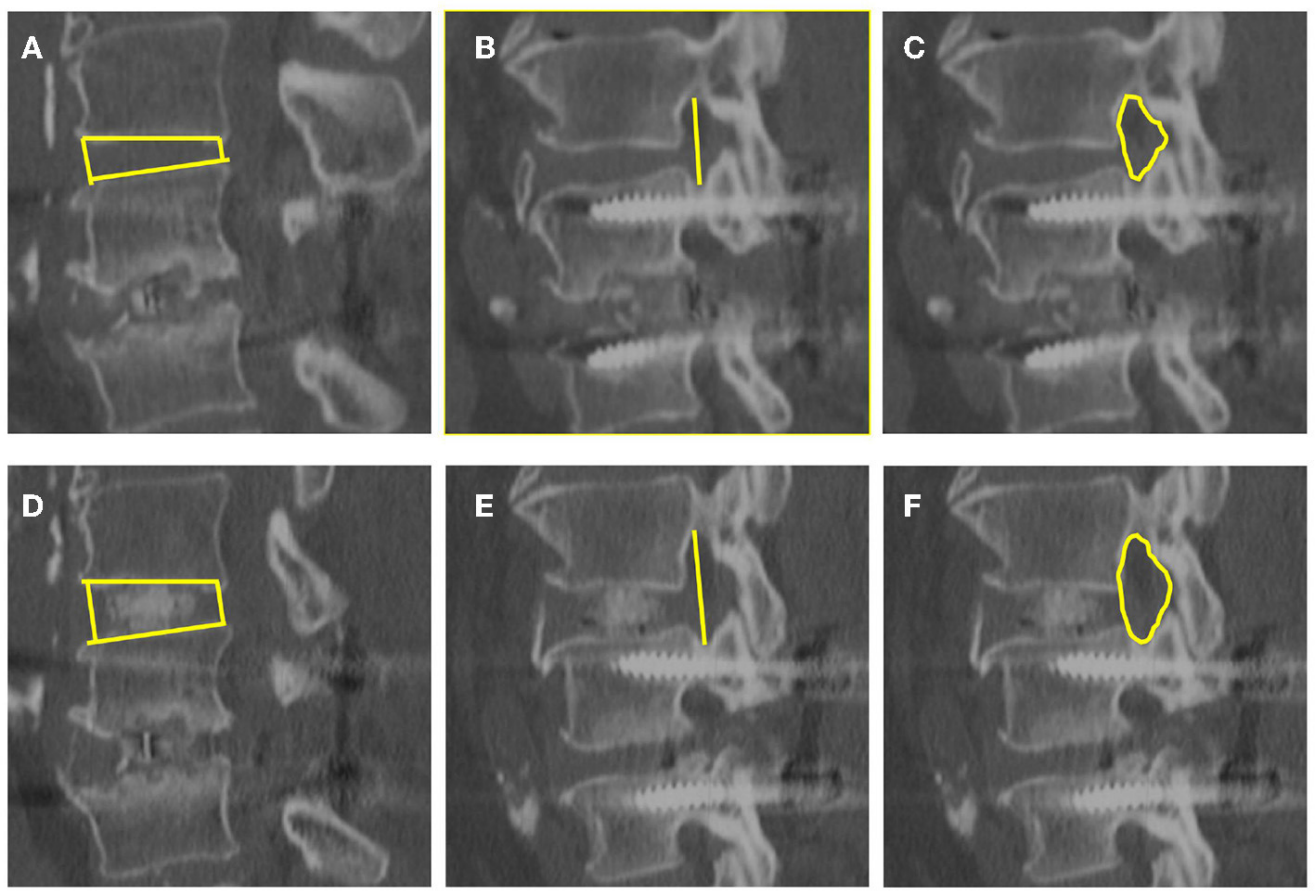
Images obtained in the same patient. Disk height **(A,D)**, foraminal height **(B,E)**, and foraminal CSA **(C,F)** were evaluated with CT before and at last follow-up after surgery. **(A–C)** are before surgery, and **(D–F)** are at last follow-up after surgery.

Back and leg pain was evaluated according to the visual analog scale (VAS). The Oswestry Disability Index (ODI) before surgery and at last routine postoperative clinic visits were also compared. Achievement of minimum clinically important difference (MCID) was evaluated using following thresholds: ODI 10, back pain VAS 2.1, leg pain VAS 2.8. Radio (22, 23) graph, Computed Tomography (CT) and Magnetic Resonance Imaging (MRI) before and at last follow-up after surgery was evaluated in all patients. Axial CSA of the spinal canal at the ASD level were evaluated by T2-weighted MRI (Figure 1). DH, height of intervertebral foramen, and CSA of intervertebral foramen were evaluated with CT (Figure 2). Averages of the anterior and posterior heights of the disk were used for disk height; and largest diameter of foramen was used for evaluation. Segmental lordosis (SL) was determined by measuring the sagittal Cobb angle between the upper endplate of the upper vertebral body in relation to the lower endplate of the lower vertebral body fused. Lumbar lordosis (LL) was determined by measuring the sagittal Cobb angle between the upper endplate of the L1 and S1 vertebra. All diameters and CSAs were measured using a picture archiving and communication systems (PACS). Grading of severity of lumbar spinal stenosis based on reports by Schizas et al. (24). Radiograph at last follow-up was used to evaluate subsidence (25). CT images obtained at last follow-up were reviewed to assess bridging bone to determine if bony fusion had occurred. Fusion criteria on CT studies included the presence of bony trabeculation across the fusion level and lack of bony lucency at the graft/vertebral body junction (26–28). Evaluation of bone fusion was blinded and performed by 3 surgeons. Fusion was identified if at least 2 of the observers concurred. Complications during surgery and follow-up periods are detected by assessment and physical examination according to past reports and patient self-reports (18, 29, 30). Data on perioperative and postoperative complications in the patients were collected and reviewed.

## Results

The 13 consecutive patients were included in this study. The mean patient age was 68.5 ± 8.7 years. The mean BMI (Body Mass Index) was 26.5 ± 3.2 Kg/m^2^. All of the cases were successfully treated with stand-alone OLIF. The mean operation time was 64.9 ± 18.9 mins. The mean blood loss was 22.3 ± 8.6 mL. Hospital stay post operation was 3.8 ± 0.8 days. The mean follow-up was 17.7 ± 8.3 months. The mean follow-up time after operation was 17.7 ± 8.3 months. Demographic and operative characteristics of the patients was shown in Table 1.

**Table 1:** Summary of patient demographics and surgical characteristics.

**Patient No**.	**Sex**	**Age (yrs)**	**BMI (Kg/m^**2**^)**	**Comorbidities**	**Prior operation**	**Time before revision (mos)**	**Symptoms before revision**	**Level**	**Radiographic findings**	**Time of operation (mins)**	**Blood loss (mL)**	**Hospital stay post revision (days)**	**Follow-up (mos)**	**Fusion**	**Subsidence**
1	M	61	24.22	HT	TLIF L4/5, L5S1	16	Low back pain, pain in bilateral lower extremities	L3/4	Spinal canal (Grade C), bilateral foraminal stenosis	45	30	4	33	Yes	No
2	M	80	29.41	HT, DM	TLIF L3/4	48	Low back pain, Intermittent claudication.	L2/3	Loss of intervertebral height, spondylolisthesis, lumbar spinal stenosis (Grade C), LDH	100	35	5	25	Yes	No
3	M	81	24.46	HT	PLIF L3/4, 4/5	156	Low back pain	L2/3	Loss of intervertebral height, spondylolisthesis, lumbar spinal stenosis (Grade A3), bilateral foraminal stenosis	105	40	4	25	Yes	Grade 1
4	F	66	24.44	CHD	TLIF L4/5	26	Pain in left lower extremity, intermittent claudication	L3/4	Lumbar spinal stenosis (Grade B), left foraminal stenosis	40	20	3	22	Yes	Grade 1
5	F	79	29.78	HT	TLIF L4/5, L5S1	104	Low back pain, pain in left lower extremity	L3/4	Lumbar spinal stenosis (Grade C), left foraminal stenosis, lumbar instability	67	25	3	26	Yes	No
6	F	64	25.07	HT, DM	TLIF L4/5	24	Pain in right lower extremity	L3/4	Loss of intervertebral height, lumbar spinal stenosis (Grade B), right foraminal stenosis, LDH	60	20	4	22	Yes	No
7	F	71	24.17	HT	PDF L3/4, 4/5	180	Low back pain, intermittent claudication	L2/3	Lumbar spinal stenosis (Grade B), lumbar instability	56	20	3	16	Yes	No
8	M	57	25.83	CHD, DM	TLIF L4/5, L5S1; Endoscopic discectomy L3/4	108	Low back pain, pain in right lower extremity, intermittent claudication	L3/4	Loss of intervertebral height, spondylolisthesis, lumbar spinal stenosis (Grade C), bilateral foraminal stenosis, effusion of facet joints	75	20	4	10	Yes	No
9	M	65	27.66	HT	TLIF L5S1	60	Low back pain, pain in right lower extremity	L4/5	Loss of intervertebral height, spondylolisthesis, lumbar spinal stenosis (Grade A3), bilateral foraminal stenosis	60	20	5	8	Yes	No
10	F	54	34.6	HT	TLIF L4/5 and PDF L5S1; Removal of implants	28	Low back pain, pain in right lower extremity, intermittent claudication	L3/4	Loss of intervertebral height, lumbar spinal stenosis (Grade D), left foraminal stenosis	65	20	3	8	Yes	No
11	M	68	25.71	HT, DM	PLIF L3/4, 4/5	41	Low back pain, pain in bilateral lower extremities	L2/3	Loss of intervertebral height, spondylolisthesis, lumbar spinal stenosis (Grade B), bilateral foraminal stenosis	60	20	3	13	Yes	No
12	M	68	22.49	HT, DM	TLIF L4/5, L5S1	52	Low back pain, pain in left lower extremity	L3-4	Loss of intervertebral height, spondylolisthesis, lumbar spinal stenosis (Grade A3), left foraminal stenosis	58	10	3	9	Yes	NO
13	M	77	26.47	HT, DM	TLIF L4/5, L5S1	144	Low back pain, pain in left lower extremity, intermittent claudication	L3/4	Loss of intervertebral height, spondylolisthesis, lumbar spinal stenosis (Grade D), left foraminal stenosis LDH	53	10	5	13	Yes	No

*CHD, Coronary heart disease; DM, Diabetes mellitus; HT, Hypertension; LDH, lumbar disc herniation; PDF, Posterior decompression and fusion; PLIF, Posterior Lumbar Interbody Fusion; TLIF, Transforaminal lumbar Interbody fusion*.

Low back pain, leg pain evaluated by VAS were significantly improved at last follow-up after surgery compared with before surgery (*P* < 0.01, Table 2). ODI was also significantly improved at last follow-up months after surgery compared with before surgery (*P* < 0.01, Table 2). The percentage attainment of MCID at last follow-up for back pain VAS, leg pain VAS and ODI was 100, 100, and 84.62%, respectively.

**Table 2:** Clinical outcomes.

**Parameter**	**Value preop**	**Value postop**	***p***
VAS, back	6.2 ± 1.0	2.0 ± 1.1	<0.01
VAS, leg	7.0 ± 1.9	1.0 ± 0.9	<0.01
ODI	28.0 ± 7.5	10.8 ± 4.0	<0.01

The DH, the axial CSA of spinal canal, the foraminal height and the CSA of foramen were significantly enlarged at last follow-up compared those before surgery (*P* < 0.01, Figure 2). Segmental lordosis improved from a mean of 5.4° ± 7.7° to 8.7° ± 4.5° (*P* < 0.05) between the preoperative and final follow-up radiographs. Global lumbar lordosis (L1–S1) increased from 34.8° ± 13.5° to 40.8° ± 10.0° (*P* < 0.05) comparing preoperative and last follow-up radiographs (Figure 2). All of the 13 patients achieved bony fusion during their follow-up period (Table 1).

**Figure 3:**
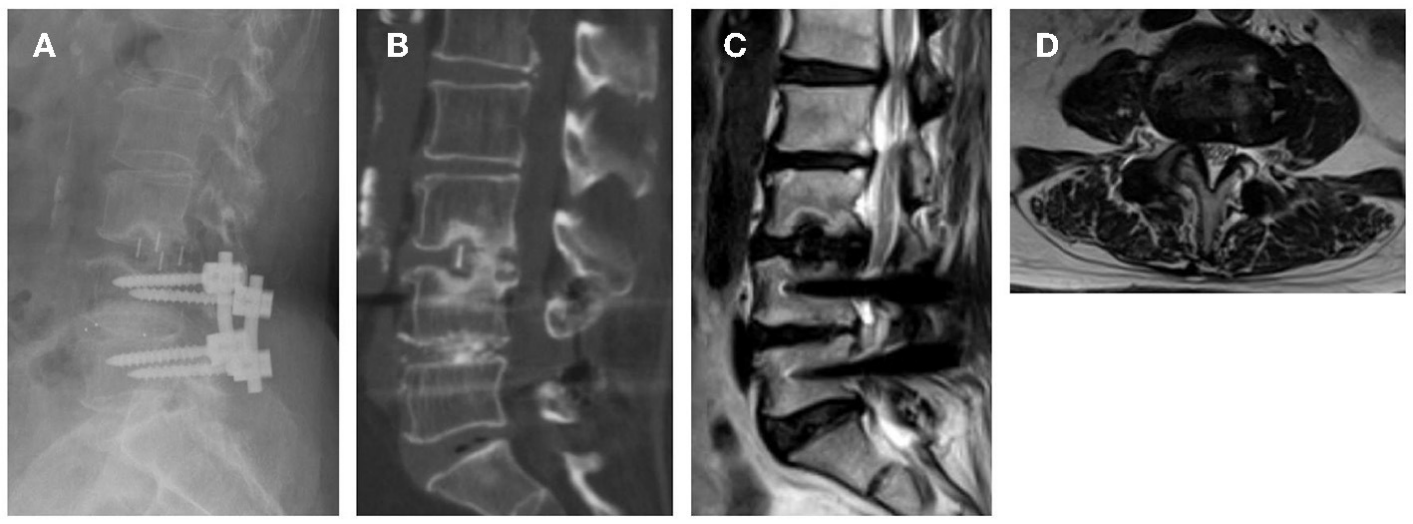
Images obtained in a 66-year-old woman who had an L4–L5 posterior instrumented fusion 26 month ago. She experienced new leg pain and intermittent claudication due to adjacent-segment degeneration and stenosis for 2 months. **(A)** 18 months follow-up reviewed Grade I subsidence; **(B)** CT confirmed bony fusion was achieved; **(C,D)** Indirect decompression of spinal canal was maintained.

Grade I cage subsidence at 2 levels was observed in 2 of the patients by their last follow-up (Figure 3). However, at their last follow-up, the clinical symptoms were significantly relieved, and improved VAS, ODI scores were achieved. The OLIF procedures in the lumbar spine are associated with transient or permanent symptoms in the thigh. Due to retrospective direction of observation, these data were not consistently available and thus were not included in the study. However, thigh numbness, pain, dysesthesias, or weakness indicative of a lumbosacral plexopathy was not seen in any of the patients by their last follow-up visit. No patients experienced infection or injuries to the great abdominal vessels, abdominal viscera and ureters.

## Discussion

Adjacent-segment degeneration is an undeniable phenomenon after lumbar fusion surgery. The incidence of clinically symptomatic ASD following lumbar fusion is about 3% annually (6, 31). This risk is increased in older patients, male patients and with pre-existing facet or disk degeneration. Additional risk factors include multi-level constructs and floating fusion (5, 32). Disruption of sagittal or coronal balance and ligamentous disruption can accelerate degeneration of adjacent segments in the lumbar spine (31, 33). Given this high incidence, spinal surgeons are facing a growing population of patients in need of treatment for ASD.

One clinical question that arises is whether or not ASD can be managed non-operatively. Within our review, no study directly compared non-operative with operative management for ASD. However, from a clinical perspective, it stands to reason that non-operative treatment would first be implemented and operative treatment undertaken only after failure of non-operative treatment, just as in primary lumbar pathology (6). However, it is unclear how effective non-operative care is, because there are no studies directly comparing non-operative care with surgical intervention for ASD. Thus, clinical judgment, best available evidence, and patient preference are the current cornerstones that guide treatment (6).

Another issue to address is the type of operative treatment to choose. ASD may be associated with kyphosis, severe disc collapse, listhesis, or hypertrophied ligamentum flavum causing adjacent segment stenosis. However, there is absence of literature directly comparing one type of operative treatment with another type.

For cases involving only neural entrapment without axial symptoms, the surgeon may choose limited decompression. A potential benefit of laminectomy alone is that minimally invasive surgery (MIS) may be performed. However, this approach risks iatrogenic destabilization adjacent to a fused construct and increases the risk of recurrent ASD (31). A more typical approach is to perform revision posterior surgery with both a laminectomy and extension of the instrumentation and fusion to the rostral levels (3, 34, 35). In the setting of symptomatic ASD with radiographic evidence, patients often have significant improvement in pain and quality of life with 2 years minimum follow up (6, 34, 36). However, revision surgery through a previous lumbar incision can be cumbersome in the setting of scar tissue and violation of natural landmarks from the initial surgery. This can lead extensive soft tissue dissection, adding to surgical blood loss, severe postoperative pain, prolonging operation time and recovery with high associated health care (8). In addition, exposing the previous laminectomy site poses a higher risk of dural violations and CSF leakage due to postoperative scar tissue (10, 37, 38).

The standard of care in ASD remains structural stabilization, or extension of the construct, and decompression (34, 35). Thus, other approach has been proposed to avoid the disadvantages. Chen et al. introduced cortical bone trajectory screws fixation with minimal invasive interbody cage fusion for lumbar adjacent segment disease to negates removal of pre-existing instruments and reduce the wound length, blood loss and soft tissue damage compared with traditional surgery (33). Minimally invasive lateral interbody fusion is another viable option for the treatment of ASD. It is an entirely different access route to the spine, which could avoid previous lumbar incision and disruption of the posterior tension band. Minimally invasive lateral interbody fusion of the adjacent segment and posterior extension of fusion could achieve clinical and radiographic improvement of ASD (33). Du et al. reported lateral lumbar interbody fusion (LLIF) with unilateral pedicle screw fixation for the treatment of ASD (39). Wang et al. reported LLIF without supplemental pedicle screw fixation and Palejwala et al. reported LLIF using a stand-alone construct for the treatment of ASD (3, 7). All of above studies shows significantly reduced pain and favorable radiographic results treated by LLIF (3, 7, 33, 39). However, the LLIF approach is associated with access-related thigh pain caused by direct muscle injury and has an added a risk of injury to the lumbar plexus (40–43). Furthermore, high rates of transient anterior thigh symptoms are found despite real-time electromyography monitoring (16). Thus, OLIF has been applied recently to avoid invasion of the psoas muscle and lumbar plexus (17, 18).

Zhu et al. compared stand-alone oblique lumbar interbody fusion with posterior lumbar interbody fusion for revision of rostral adjacent segment disease and concluded that OLIF was effective and safe for the treatment of rostral ASD following prior posterior lumbar fusion, and is superior to PLIF in terms of perioperative parameters, short-term clinical outcomes, and DH restoration, with similar fusion and reduction rates (21). In our study, we reviewed a consecutive series of patients who had undergone OLIF using a stand-alone construct for the treatment of ASD. This approach achieved satisfied clinical outcomes (Table 2). It also achieved radiographic indirect decompression by enlarging the DH, the axial CSA of spinal canal, the foraminal height and the CSA of foramen (Figure 2). Besides, it showed the advantage of being minimally invasive. The mean blood loss of 28.3 ± 8.2 ml, the mean operation time of 69.5 ± 27.4 min and the mean hospital stay of 3.8 ± 0.8 days demonstrate that this approach is likely to cause less morbidity than a posterior approach to the spine. There is no need to involve a laminectomy and extension of instrumentation as a standard posterior revision surgery do. Thus, CSF leakage and the management of previously implanted spinal instrumentation could be avoided. Another advantage of this approach is that the posterior spinal elements, including the facet joint capsules, are not disrupted; thus, additional degeneration at the supra adjacent level may also be less likely to occur (3). Moreover, this procedure improves SL and global LL after lateral surgery. Thus, its application in patients may have potential benefits for sagittal imbalance.

A major concern regarding the use of stand-alone OLIF is that the construct may not be strong enough to promote fusion, and prevent the interbody cages from subsidence. As we all know, indirect neural decompression in OLIF was achieved by reduction of disc bulging and elongation of the hypertrophied ligamentum flavum through the restoration of DH (12). Cage subsidence caused by endplate damage, improper cage size, and osteoporosis may affect indirect neural decompression and interbody fusion. However, low-grade subsidence is likely an expected outcome, while high-grade subsidence may result in persistent symptoms or reoperation (25, 44). Moreover, stand-alone construct has shown evidence of solid arthrodesis and improvements in clinical symptoms and fusion rate was not affected by incidence of subsidence (25, 45–47). Careful attention to proper endplate preparation without violation of the cortical endplate is also critical to minimizing settling (3). In our study, Grade I cage subsidence was observed in 2 of the patients by their last follow-up. However, the bony fusion was achieved and the clinical symptoms were improved as well. Therefore, we believe indirect decompression could be maintain as long as bony fusion was achieved and high-grade subsidence was avoided.

There are several limitations to this study. With regard to the relief of neurological symptoms of stenosis, the OLIF approach relies entirely on indirect decompression by elevating intervertebral disc height, which expands the neuroforamen and tensions the ligaments to open the central canal. While we found excellent clinical and radiographic results, one might expect this approach to be occasionally inadequate in cases of severe stenosis, as well as in cases without severe disc collapse. A larger study with a wider variety of specific pathologies would be helpful to validate this technique across the broad spectrum of ASD. Another limitation relates to our ability to ascertain definitive fusion. In this series, we used reconstructed CT scans to identify bridging bone between the treated vertebral bodies as the determinant of fusion. However, a follow-up longer than 2 years would also be helpful as well, as an osseous nonunion would likely become more apparent clinically or radiographically over more protracted periods of time.

## Conclusion

This limited study suggests that OLIF using a stand-alone construct may be a safe and effective alternative way in treating ASD following a previous lumbar fusion. The approach can achieve adequate indirect neural decompression, satisfied clinical outcome as well as solid arthrodesis without supplemental fixation.

## Data Availability Statement

The original contributions presented in the study are included in the article/supplementary material, further inquiries can be directed to the corresponding author/s.

## Ethics Statement

The studies involving human participants were reviewed and approved by the Ethics Committee of Xuanwu Hospital of Capital Medical University. The patients/participants provided their written informed consent to participate in this study. Written informed consent was obtained from the individual(s) for the publication of any potentially identifiable images or data included in this article.

## Author Contributions

WK is responsible for the writing of the paper. CC is responsible for the search for data and the inclusion of cases. QY is responsible for the design of the study. CZ is responsible for the evaluation of the results. FJ is responsible for the statistics of the data. HW is the instructor of the entire study. All authors contributed to the article and approved the submitted version.

## Funding

This research was supported by the Beijing Natural Science Foundation of China, the Beijing Natural Science Foundation of China Joint Project (KZ20191025028).

## Conflict of Interest

The authors declare that the research was conducted in the absence of any commercial or financial relationships that could be construed as a potential conflict of interest.

## Publisher's Note

All claims expressed in this article are solely those of the authors and do not necessarily represent those of their affiliated organizations, or those of the publisher, the editors and the reviewers. Any product that may be evaluated in this article, or claim that may be made by its manufacturer, is not guaranteed or endorsed by the publisher.
